# Sex-specific AKI risk in acute myocardial infarction patients with type 2 diabetes mellitus

**DOI:** 10.3389/fendo.2025.1654587

**Published:** 2025-09-30

**Authors:** Xiaorui Huang, Haichen Wang, Wei Yuan

**Affiliations:** ^1^ Department of Cardiovascular Medicine, The First Affiliated Hospital of Xi’an Jiaotong University, Xi’an,, China; ^2^ Department of Cardiovascular Surgery, The First Affiliated Hospital of Xi’an Jiaotong University, Xi’an,, China

**Keywords:** acute myocardial infarction (AMI), acute kidney injury (AKI), type 2 diabetes mellitus (T2DM), sex-specific, risk factor

## Abstract

**Background/objectives:**

While sex differences in cardiovascular outcomes are recognized, their role in the risk and clinical outcomes of acute kidney injury (AKI) among acute myocardial infarction (AMI) comorbid with type 2 diabetes mellitus (T2DM) remains unstratified in clinical guidelines. The aim of this study is to explore the sex differences in the occurrence of AKI among AMI-T2DM patients, so as to provide ideas for the precision management of these patients.

**Methods:**

This retrospective cohort study enrolled AMI patients with T2DM from The First Affiliated Hospital of Xi’an Jiaotong University from 2018 to 2022. Clinical data and medication information were collected through the hospital’s biospecimen information resource center. Patients enrolled were divided into male group and female group. The primary outcome is AKI during hospitalization.

**Results:**

Among 2,631 AMI patients complicated with T2DM (76.1% male, median age 67.0 years (55.9–78.1), acute kidney injury occurred in 13.3% (n = 351) of the cohort. It shows higher AKI incidence in females (17.2% vs. 12.1%, P = 0.026) with distinct sex-specific risks: Higher HbA1c was paradoxically protective in both sexes (female OR = 0.73; male OR = 0.81), hyperkalemia impact (OR = 5.88 vs. 4.02), and HDL protection (OR = 0.16); males exhibited hyperphosphatemia hazard(OR = 14.32). STEMI unexpectedly reduced AKI risk in both sexes (female OR = 0.36; male OR = 0.64). Univariate regression analysis shows the association between electrolyte imbalances, particularly hyperphosphatemia, and AKI risk was significantly stronger in males (OR = 14.3) than in females (OR = 5.2). Conversely, abnormalities in lipid metabolism demonstrated a significant protective effect against AKI exclusively in females. Additionally, advanced age, higher Killip class, hypoalbuminemia, and elevated fibrinogen were significant predictors of AKI development in both sexes.

**Conclusions:**

This study reveals significant sex disparities in AKI risk among T2DM-AMI patients: females show higher incidence, while hyperphosphatemia strongly predicts risk in males and hyperkalemia/Killip class in females. Elevated HbA1c paradoxically reduced risk in both. We recommend sex-specific management: monitor phosphorus in males and potassium with hemodynamics in females. Future work should develop sex-stratified risk models and clarify mechanisms.

## 1 Introduction

Acute myocardial infarction (AMI) and type 2 diabetes mellitus (T2DM) present major threats to global public health. The Global Burden of Disease Study (1) reports roughly 15 million new AMI cases globally each year, with 30%-40% involving comorbid T2DM. This rate surpasses 45% in East Asian populations. Critically, in-hospital mortality for AMI patients with T2DM is 1.8 times higher than for those without diabetes (2). Acute kidney injury (AKI) incidence rises sharply during AMI in these patients, further elevating mortality to 40%-60% (3, 4), concentrating heavy burdens on individuals and society.

AKI diagnosis follows the 2012 KDIGO criteria (5). It is a strong independent predictor of in-hospital mortality for AMI patients and linked to worse short- and long-term outcomes (6, 7). A Mayo Clinic study of 9,199 AMI patients found AKI significantly increased in-hospital mortality (11.1% vs 1.0%, *P* < 0.001; adjusted HR 5.75; 95% CI 4.06-8.13) and long-term adverse events among survivors (8). Alarmingly, T2DM impairs renal repair; roughly 30% of AKI survivors develop chronic kidney disease (CKD) (9, 10). While KDIGO guidelines advise AKI monitoring in high-risk groups, current risk prediction models—such as the Mehran Risk Score—exhibit limited predictive performance specifically in the T2DM-AMI population (11–13). Critically, these models fail to adequately account for sex-specific pathophysiology, as evidenced by their lack of sex-interaction terms and consistent underperformance in female patients, leading to notable underdiagnosis and suboptimal management in women. Current AKI prevention and treatment strategies predominantly use “sex-neutral” approaches, overlooking potential sex-specific factors. This gap means some patients miss out on intervention benefits. While a few studies suggest sex may affect AKI risk in AMI patients, this remains unconfirmed in those with comorbid T2DM.

Biological mechanisms underpinning sex differences in cardiorenal disease are increasingly recognized (14). Estrogen-mediated endothelial protection and androgen-driven profibrotic pathways have been implicated in differential AKI susceptibility (15, 16). Yet, most contemporary studies treat sex as a confounding variable rather than a key biological determinant, and its interaction with AMI-induced AKI in the context of T2DM remains poorly quantified. Therefore, the objective of this study is to investigate sex-specific risk factors and differential AKI susceptibility in patients with comorbid T2DM and AMI. To address this gap, we will recruit first-time AMI patients with T2DM from the First Affiliated Hospital of Xi’an Jiaotong University. By collecting detailed renal and clinical parameters, we aim to elucidate sex-based disparities in AKI risk and provide evidence supporting sex-stratified precision management strategies.

## 2 Materials and methods

### 2.1 Study population

This study aimed at conducting a retrospective analysis of AMI patients combined with T2DM and admitted in the First Affiliated Hospital of Xi’an Jiaotong University from January 2018 to December 2022. This study set these inclusion criteria: 1) meeting the diagnostic criteria for AMI and T2DM; 2) underwent renal function testing during admission and complete clinical data. The exclusion criteria were as follows: 1) Patients with pre-existing chronic kidney diseases, previously diagnosed as diabetic nephropathy, chronic kidney disease, chronic nephritis, etc.; 2) Patients comorbid with malignancies, chronic wasting diseases, psychiatric disorders, or autoimmune diseases; 3) Patients with incomplete clinical data. Patients were divided into male group and female group.

This study was conducted in accordance with the principles outlined in the Declaration of Helsinki. The study protocol was reviewed and granted ethical approval by the Institutional Review Board of Xi’an Jiaotong University (approval number: XJTU1AF2025LSYY-433). Due to the retrospective and observational nature of this study, which involved only the analysis of existing, de-identified clinical data, the requirement for obtaining individual informed consent was waived by the ethics committee. All patient data were handled in strict compliance with institutional guidelines to ensure confidentiality and privacy.

### 2.2 Definitions and outcome

**Figure 1:**
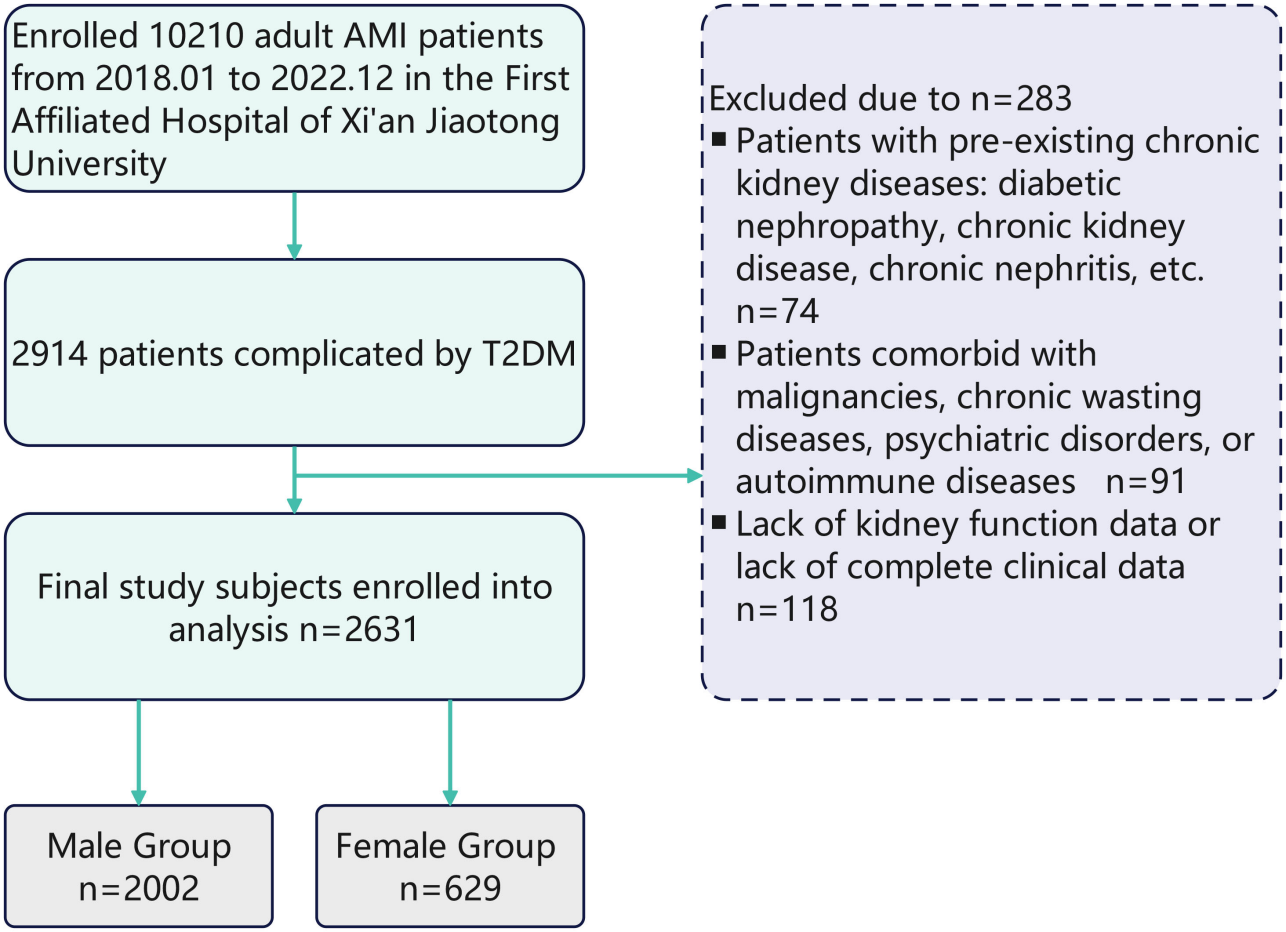
Flowchart of sex-stratified AKI risk assessment in T2DM-AMI patients. From January 2018 to December 2022, 10210 adult AMI patients were enrolled from the First Affiliated Hospital of Xi’an Jiaotong University. Among them, 2914 patients were complicated by T2DM. After excluding 283 patients due to pre - existing chronic kidney diseases (n = 74), comorbidities with malignancies, chronic wasting diseases, psychiatric disorders, or autoimmune diseases (n = 91), and lack of kidney function data or complete clinical data (n = 118), 2631 patients were finally included in the study analysis. These patients were divided into a male group (n = 2002) and a female group (n = 629).

**Figure 2:**
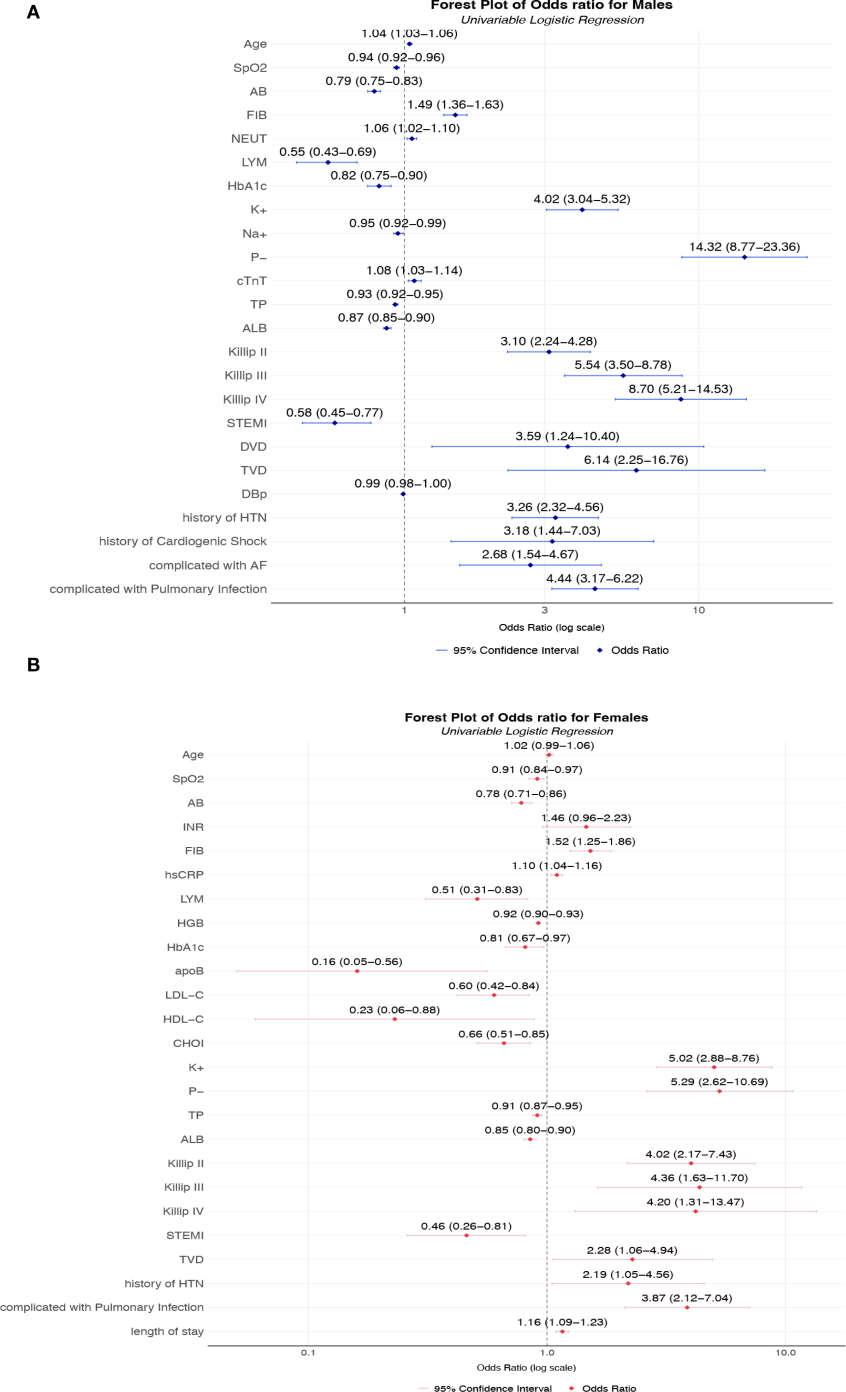
**(A)** Unadjusted odds ratios for AKI risk factors in male T2DM-AMI patients; **(B)** Unadjusted odds ratios for AKI risk factors in female T2DM-AMI patients. **(A)** Univariable logistic regression analysis of male patients with AMI and T2DM (n = 2002). Squares represent point estimates (ORs), horizontal lines indicate 95% confidence intervals, and the vertical dashed line denotes the null effect (OR = 1). Key observations: The highest-risk factors included hypophosphatemia (P^−^, OR = 14.32, 95% CI 8.77-23.36), Killip class IV (OR = 8.70, 95% CI 5.21-14.53), and triple-vessel disease (TVD, OR = 6.14, 95% CI 2.25-16.76. Protective factors were lymphocyte (LYM, OR = 0.55, 95% CI 0.43-0.69), serum albumin (ALB, OR = 0.87, 95% CI 0.85-0.90), and oxygen saturation (SpO_2_, OR = 0.94, 95% CI 0.92-0.96). In terms of electrolyte disorders, hyperkalemia (K^+^) significantly increased the risk (OR = 4.02, 95% CI 3.04-5.32), while hyponatremia (Na^+^) showed a protective effect (OR = 0.95, 95% CI 0.92-0.99). **(B)** Univariable logistic regression analysis of female patients with AMI and T2DM (n = 629). Squares represent point estimates (ORs), horizontal lines indicate 95% confidence intervals, and the vertical dashed line denotes the null effect (OR = 1).Key findings: Hyperkalemia (K^+^, OR = 5.02, 95% CI 2.88-8.76) and hypophosphatemia (P^−^, OR = 5.29, 95% CI 2.62-10.69) were primary risk factors, with pulmonary infection (OR = 3.87, 95% CI 2.12-7.04) and Killip class II (OR = 4.02, 95% CI 2.17-7.43) significantly elevating risk. Apolipoprotein B (apoB, OR = 0.16, 95% CI 0.05-0.56) and high-density lipoprotein (HDL-C, OR = 0.23, 95% CI 0.06-0.88) demonstrated strong protection, while lymphocyte count (LYM, OR = 0.51, 95% CI 0.31-0.83) and Actual Bicarbonate (AB, OR = 0.78, 80% CI 0.71-0.86) reduced risk. *Univariable analysis without covariate adjustment, reflecting crude association strength. SpO_2_, Peripheral Oxygen Saturation; AB, Actual Bicarbonate; INR, International Normalized Ratio; FIB, Fibrinogen; hsCRP, High-Sensitivity C-Reactive Protein; LYM, Lymphocyte count; HGB, Hemoglobin; Hb1Ac:Hemoglobin A1c; apoB, Apolipoprotein B; LDL-C, Low-Density Lipoprotein Cholesterol; HDL-C, High-Density Lipoprotein Cholesterol; CHOI, Total Cholesterol; K^+^, Potassium ion; P-, Inorganic Phosphate; TP, total protein; ALB, Albumin; DVD:double-vessel disease TVD, triple-vessel disease; HTN, Hypertension; LVEF, left ventricular ejection fraction.

Data of clinical characteristics, lab tests and coronary angiography conclusion are obtained from the hospital’s unified clinical database, which is managed and integrated by the institutional Biobank Department. The diagnoses of AMI and T2DM are based on the latest guidelines currently available. According to the KDIGO 2012 criteria (5), AKI is defined as:An increase in serum creatinine (SCr) by ≥0.3 mg/dL (≥26.5 μmol/L) within 48 hours, ≥ 1.5-fold increase in SCr from baseline, or urine output < 0.5 mL/(kg·h) for ≥ 6 hours. Baseline Scr was defined as the lowest value within 3 months prior to admission. AKI is diagnosed if any of these criteria are met without known cause for previous kidney injury. The stage of AKI is shown in **Table 1**. The primary outcome is the development of acute kidney injury during hospitalization. **Figure 1** shows the flow chart of the study **Figure 2**.

**Table 1:** Diagnostic and staging criteria for AKI.

Stage	Serum creatinine criteria	Urine output criteria
1	Increase ≥ 0.3 mg/dL (26.5 μmol/L) within 48h or 1.5–1.9x baseline	< 0.5 mL/kg/h for 6–12 hours
2	2.0–2.9x baseline	< 0.5 mL/kg/h for ≥ 12 hours
3	Increase ≥ 4.0 mg/dL(353.6 μmol/L) or 3.0x baseline	< 0.3 mL/kg/h for ≥ 24 hours OR Anuria for ≥ 12 hours

Diagnostic criteria are based on the Kidney Disease: Improving Global Outcomes (KDIGO) Clinical Practice Guideline for Acute Kidney Injury (5).

### 2.3 Statistical analysis

Continuous variables were summarized as mean ± standard deviation if normally distributed or as median with interquartile range (IQR) if non-normally distributed, based on visual inspection and the Shapiro-Wilk test for normality. For ordered categorical variables between two groups, the Mann-Whitney U test was used for comparison; for unordered multicategorical variables between two groups, the Chi-square test was applied. Univariate and multivariate logistic regression analyses were used to test the association between predictive indicators and AKI. All candidate variables with potential relevance to the outcome (e.g., demographic, clinical, and laboratory factors) are included in the univariate analysis. Each variable undergoes separate logistic regression to assess its crude association with the outcome, yielding OR, 95% CI, and p-value. Variables with *P* < 0.1 in univariate analysis or clinical relevance were included in multivariable models. A multivariate Cox proportional hazards regression model was used to assess the independent associations between factors (including age, potassium ion level, glycated hemoglobin, presence of STEMI, cardiac function classification, and history of hypertension) and the risk of AKI. The model was adjusted for known clinically important confounding factors, including age, history of hypertension, heart failure, and left ventricular ejection fraction (LVEF, included as a continuous variable). Results are presented as adjusted ORs with their corresponding95% CIs. Categorical variables were expressed as percentages N (%), and comparisons between groups were made using the χ² test. Data analyses were performed using the SPSS (version 26.0 package (IBM, Armonk, NY, USA). Figures were plotted using GraphPad Prism 9.0 (GraphPad Prism Software Inc., San Diego, CA, USA). *P* < 0.05 was considered statistically significant.

## 3 Results

### 3.1 Baseline characteristics and clinical outcomes

A cohort of 2,631 diabetic patients presenting with acute myocardial infarction were enrolled, comprising 2,002 males (76.1%) and 629 females (23.9%). A total of 351(13.3%) patients developed AKI. Significant sex-based disparities were observed across multiple clinical domains. Significantly higher acute kidney injury incidence was recorded among females (17.2% vs. 12.1%, *P* = 0.001), with differential severity distribution across KDIGO stages: stage 1 AKI was observed in 8.4% of females versus 5.8% of males (*P* = 0.025), while stages 2–3 AKI were documented in 8.7% of females versus 6.3% of males (*P* = 0.045). Female patients were characterized by advanced age (72.4 ± 9.5 vs. 65.3 ± 11.1, *P* < 0.001), longer diabetes duration (9.4 ± 6.7 vs. 7.9 ± 5.8, *P* < 0.001), and poorer glycemic control as evidenced by elevated HbA1c (8.31 ± 1.85 vs. 8.13 ± 1.70, *P* = 0.012) and higher random blood glucose levels (11.44 ± 4.98 vs. 10.82 ± 4.59, *P* = 0.023). Higher comorbidity burdens were documented in females, with significantly greater hypertension prevalence (71.7% vs. 60.4%; *P* < 0.001) and stroke history (16.9% vs. 11.7%, *P* = 0.001). Comparable ventricular function parameters were observed between sexes, with no statistically significant differences identified in left ventricular ejection fraction, ventricular dimensions, or blood pressure metrics (**Table 2A**). Divergent arrhythmia patterns were documented, with females exhibiting higher overall malignant arrhythmia incidence (12.4% vs. 7.6%, *P* < 0.001) and demonstrated elevated rates of atrial fibrillation (7.0% vs. 3.4%, *P* < 0.001). Hospitalization complications revealed substantially higher pulmonary infection rates in females (14.6% vs. 9.7%, P = 0.001), along with increased ventricular aneurysm occurrence (5.1% vs. 3.2%, *P* = 0.033).

Procedural variations were noted, with coronary artery bypass grafting more frequently performed in females (4.5% vs. 1.4%), though percutaneous coronary intervention rates were comparable between groups. Mortality risk stratification by Killip classification revealed equivalent distributions of high-risk categories (Class III-IV: 8.6% both sexes). These comprehensive findings demonstrate significant sex-specific clinical trajectories in T2DM-AMI patients, with females exhibiting heightened susceptibility to metabolic dysregulation, renal impairment, and infectious complications requiring tailored therapeutic approaches (**Tables 2A**, **B**).

**Table 2A:** Baseline characteristics and comorbidities of study patients with T2DM and AMI, stratified by sex.

Variable	Overall (n = 2631)	Male (n = 2002)	Female (n = 629)	*P*
age	67.0 ± 11.1	65.3 ± 11.1	72.4 ± 9.5	< 0.001
Diabetes				
Duration, year	8.5 ± 6.2	7.9 ± 5.8	9.4 ± 6.7	0.000
Random blood sugar, mmol/L	10.96 ± 4.69	10.82 ± 4.59	11.44 ± 4.98	0.004
HbA1c (%)	8.17 ± 1.74	8.13 ± 1.70	8.31 ± 1.85	0.027
Medical History				
History of HTN, n (%)	1660 (63.1%)	1209 (60.4%)	451 (71.7%)	< 0.001
History of Heart Disease, n (%)	53 (2.0%)	34 (1.7%)	19 (3.0%)	0.055
History of Stroke, n (%)	341 (13.0%)	235 (11.7%)	106 (16.9%)	0.001
Cardiovascular Parameters				
HR,/min	80.0 ± 16.0	79.0 ± 15.6	80.0 ± 16.2	0.234
Avg SBp	128.23 ± 22.30	127.41 ± 21.65	130.84 ± 24.07	0.001
Avg DBp	78.45 ± 13.70	79.13 ± 13.48	76.29 ± 14.15	< 0.001
LVEF(%)	50.2 ± 10.8	50.8 ± 11.5	49.8 ± 11.4	0.218
LVESD (mm)	38.1 ± 7.2	38.1 ± 7.3	38.2 ± 7.4	0.366
LVSDD (mm)	51.6 ± 7.0	51.9 ± 7.7	51.4 ± 6.8	0.440
Angiographic Findings				
Lesion vessels				0.572
1	205 (7.8%)	156 (7.8%)	49 (7.8%)	
2	453 (17.2%)	336 (16.8%)	117 (18.6%)	
3	1973 (75.0%)	1510 (75.4%)	463 (73.6%)	
Admission Status				
STEMI, n (%)	1460 (55.5%)	1125 (56.2%)	335 (53.3%)	0.196
Killip Class				0.137
I	1914 (72.7%)	1476 (73.7%)	438 (69.6%)	
II	490 (18.6%)	353 (17.6%)	137 (21.8%)	
III-IV	227 (8.6%)	173 (8.6%)	54 (8.6%)	0.002
Kidney Outcomes				
AKI, n (%)	351 (13.3%)	243 (12.1%)	108 (17.2%)	0.026
KDIGO stage, n (%)				
1	169 (6.4%)	116 (5.8%)	53 (8.4%)	0.025
2-3	182 (6.9%)	127 (6.3%)	55 (8.7%)	0.045

Data are presented as mean ± standard deviation or n (%).

HTN, Hypertension; HR, Heart Rate; Avg SBP, Average Systolic Blood Pressure; Avg DBP, Average Diastolic Blood Pressure; LVEF, Left Ventricular Ejection Fraction; LVESD, Left Ventricular End-Systolic Dimension; LVSDD, Left Ventricular End-Diastolic Dimension; STEMI, ST-Elevation Myocardial Infarction; PCI, Percutaneous Coronary Intervention; PTCA, Percutaneous Transluminal Coronary Angioplasty; CABG, Coronary Artery Bypass Grafting.

**Table 2B:** In-hospital management, complications, and clinical outcomes of study patients with T2DM and AMI, stratified by sex.

Variable	Overall (n = 2631)	Male (n = 2002)	Female (n = 629)	*P*
In-Hospital Management, n (%)				
Treatment				0.427
Medicine	45 (1.7%)	31 (1.5%)	14 (2.2%)	
PCI/PTCA	2530 (96.2%)	1943 (97.1%)	587 (93.3%)	
CABG	56 (2.1%)	28 (1.4%)	28 (4.5%)	
Other Outcomes during Hospitalization				
Malignant arrhythmia	231 (8.8%)	153 (7.6%)	78 (12.4%)	< 0.001
Ventricular Fibrillation	70 (2.7%)	50 (2.5%)	20 (3.2%)	0.354
Atrial Fibrillation	113 (4.3%)	69 (3.4%)	44 (7.0%)	< 0.001
Cardiogenic Shock	40 (1.5%)	30 (1.5%)	10 (1.6%)	0.870
Ventricular Aneurysm	97 (3.7%)	65 (3.2%)	32 (5.1%)	0.033
Hydropericardium	3 (0.1%)	1 (0)	2 (0.3%)	0.082
Lung Infectious	287 (10.9%)	195 (9.7%)	92 (14.6%)	0.001
Intracardiac Thrombus	25 (1.0%)	20 (1.0%)	5 (0.8%)	0.645
Clinical Outcomes				
Kidney Outcomes				
AKI, n (%)	351 (13.3%)	243 (12.1%)	108 (17.2%)	0.026
KDIGO stage, n (%)				
1	169 (6.4%)	116 (5.8%)	53 (8.4%)	0.025
2-3	182 (6.9%)	127 (6.3%)	55 (8.7%)	0.045
Length of Stay, days	4.52 (2.82, 6.84)	4.58 (2.82, 6.93)	4.32 (2.77, 6.62)	0.423

Data are presented as median (interquartile range) or n (%).

PCI, Percutaneous Coronary Intervention; PTCA, Percutaneous Transluminal Coronary Angioplasty; CABG, Coronary Artery Bypass Grafting.

### 3.2 Laboratory parameters and biomarker profiles

**Table 3** presents the comprehensive laboratory and biomarker analyses, revealing significant sex-based differences in metabolic, cardiac, inflammatory, and coagulation profiles. Females exhibited higher LDL-C (2.51 ± 1.01 vs. 2.31 ± 0.86, *P* < 0.001), HDL-C (1.01 ± 0.24 vs. 0.87 ± 0.19, *P* < 0.001), and apoB (0.85 ± 0.26 vs. 0.81 ± 0.23, *P* < 0.001) compared to males. Cardiac biomarker analyses showed females had significantly elevated NT-proBNP (median: 1,700.00 vs. 664.00, *P* < 0.001), while no sex differences existed in cTnT (*P* = 0.705) or CK-MB (*P* = 0.270). Males demonstrated higher baseline CK levels (216.00 vs. 191.00 U/L; *P* = 0.027). Renal function diverged markedly: **f**emales had lower eGFR (79.62 ± 26.26 vs. 89.22 ± 25.79, *P* < 0.001) and creatinine (median: 56.00 vs. 67.00, *P* < 0.001), despite comparable BUN (*P* = 0.604). Electrolyte imbalances included higher phosphate in females (1.09 ± 0.32 vs. 0.99 ± 0.30, *P* < 0.001) and marginally elevated calcium (2.26 ± 0.17 vs. 2.24 ± 0.16, *P* = 0.013), with no sex differences in magnesium (*P* = 0.410) or potassium (*P* = 0.471). Coagulation profiles showed no significant sex difference in fibrinogen (*P* = 0.200), though females had higher D-dimer (median: 0.64 vs. 0.50, *P* = 0.011).

**Table 3:** Laboratory parameters and biomarker profiles.

Variable	Overall (n = 2631)	Male (n = 2002)	Female (n = 629)	*P*
HGB, g/L	137.05 ± 20.45	141.04 ± 19.55	124.36 ± 17.96	< 0.001
WBC, ×10^9^/L	9.26 ± 3.52	9.29 ± 3.56	9.15 ± 3.39	0.372
AB, mmol/L	22.79 ± 3.05	22.84 ± 2.99	22.60 ± 3.25	0.030
pH	7.40 ± 0.06	7.40 ± 0.05	7.41 ± 0.06	0.256
SpO2, %	94.97 ± 4.99	94.86 ± 5.38	95.32 ± 3.42	0.078
PO2, mmHg	86.23 ± 25.00	86.17 ± 25.56	86.43 ± 23.17	0.840
PCO2, mmHg	36.89 ± 5.63	37.09 ± 5.76	36.23 ± 5.14	0.003
LDL-C, mmol/L	2.36 ± 0.91	2.31 ± 0.86	2.51 ± 1.01	< 0.001
HDL-C, mmol/L	0.90 ± 0.21	0.87 ± 0.19	1.01 ± 0.24	< 0.001
apoB, g/L	0.82 ± 0.24	0.81 ± 0.23	0.85 ± 0.26	< 0.001
cTnT, ng/mL	0.35 (0.08, 1.19)	0.34 (0.07, 1.20)	0.36 (0.09, 1.15)	0.705
NT-proBNP, pg/mL	822.00 (263.60, 2382.50)	664.00 (222.60, 1899.00)	1700.00 (553.40, 4013.75)	< 0.001
CK-MB, U/L	24.35 (14.00, 70.00)	24.00 (14.00, 71.00)	27.00 (15.00, 66.00)	0.270
CK, U/L	212.00 (90.00, 657.50)	216.00 (94.00, 686.00)	191.00 (77.00, 602.75)	0.027
LDH, U/L	273.00 (216.00, 395.75)	270.00 (214.00, 398.75)	286.50 (221.25, 389.75)	0.977
AST, U/L	38.00 (23.00, 82.00)	37.00 (23.00, 82.00)	39.50 (24.00, 82.00)	0.424
ALT, U/L	30.00 (20.00, 46.00)	32.00 (21.00,47.00)	26.00 (18.00, 40.00)	0.262
eGFR, mL/min/1.73m²	86.98 ± 26.21	89.22 ± 25.79	79.62 ± 26.26	< 0.001
BUN, mmol/L	6.81 ± 3.55	6.83 ± 3.60	6.75 ± 3.38	0.604
CRE, μmol/L	65.00 (53.00, 82.00)	67.00 (56.00, 84.00)	56.00 (44.00, 74.00)	< 0.001
ALB, g/L	37.71 ± 5.16	37.85 ± 5.09	37.28 ± 5.38	0.021
Mg^2+^, mmol/L	0.97 ± 0.14	0.97 ± 0.13	0.98 ± 0.17	0.410
Ca^2+^, mmol/L	2.24 ± 0.16	2.24 ± 0.16	2.26 ± 0.17	0.013
K^+^, mmol/L	4.04 ± 0.48	4.04 ± 0.48	4.02 ± 0.50	0.471
P^-^, mmol/L	1.01 ± 0.31	0.99 ± 0.30	1.09 ± 0.32	< 0.001
FIB, g/L	3.76 ± 1.39	3.71 ± 1.40	3.93 ± 1.34	0.200
D-D, mg/L	1.26 ± 4.30	0.50 (0.29, 0.86)	0.64 (0.37, 1.11)	0.011
hs-CRP, mg/L	7.17 ± 5.96	7.03 ± 6.03	7.61 ± 5.74	0.001

Continuous data are presented as mean ± standard deviation (SD) for normally distributed variables or median with interquartile range (IQR) for non-normally distributed variables. Normality was assessed using the Shapiro-Wilk test.

CK-MB, Creatine Kinase-MB Isoenzyme; CK, Creatine Kinase; cTnT, Cardiac Troponin T; NT-proBNP, N-terminal pro-B-type Natriuretic Peptide; hs-CRP, High-Sensitivity C-Reactive Protein; LDL-C, Low-Density Lipoprotein Cholesterol; HDL-C, High-Density Lipoprotein Cholesterol; HbA1c, Hemoglobin A1c (Glycated Hemoglobin); Glu, Glucose; LDH, Lactate Dehydrogenase; CO_2_, Carbon Dioxide; Na^+^, Sodium; K^+^, Potassium; Mg;^2+^, Magnesium; D-D, D-dimer; apoB, Apolipoprotein B; AST, Aspartate Aminotransferase; ALT, Alanine Aminotransferase; HGB, Hemoglobin; eGFR, Estimated Glomerular Filtration Rate; AB, Actual Bicarbonate; PH, Potential of Hydrogen; PO2, Partial Pressure of Oxygen; PCO2, Partial Pressure of Carbon Dioxide; BUN, Blood Urea Nitrogen; CRE, Creatinine.

### 3.3 Unitivariable analysis of AKI risk factors

In males with T2DM and AMI, several factors demonstrated strong associations with AKI development. Advanced age increased AKI risk (OR = 1.04, 95% CI: 1.03-1.06). Electrolyte imbalances exhibited particularly pronounced effects: hyperphosphatemia (P^-^: OR = 14.32, 95% CI: 8.77-23.36) and hyperkalemia (K^+^: OR = 4.02, 95% CI: 3.04-5.32) emerged as potent risk factors. Hemodynamic and inflammatory markers were similarly influential, with elevated fibrinogen (FIB: OR = 1.49, 95% CI: 1.36-1.63) and reduced albumin (ALB: OR = 0.87, 95% CI: 0.85-0.90) significantly predicting AKI. Clinical severity indicators showed striking dose-response relationships: higher Killip classes (II-IV) conferred progressively elevated risks (OR = 3.10 to 8.70), while cardiogenic shock (OR = 3.18) and pulmonary infections (OR = 4.44) substantially increased susceptibility.

Among females, distinct patterns were observed. Electrolyte disturbances remained critical, with hyperkalemia (K^+^: OR = 5.02, 95% CI: 2.88-8.76) and hyperphosphatemia (P^-^: OR = 5.29, 95% CI: 2.62-10.69) demonstrating strong effects, though attenuated compared to males. Lipid metabolism abnormalities unexpectedly emerged as protective factors: decreased LDL-C (OR = 0.60, 95% CI: 0.42-0.84) and HDL-C (OR = 0.23, 95% CI: 0.06-0.88) were associated with reduced AKI risk. As in males, Killip classification powerfully predicted outcomes (OR = 4.02-4.36 for classes II-IV), while hypoalbuminemia (OR = 0.85, 95% CI: 0.80-0.90) and prolonged hospitalization (OR = 1.16, 95% CI: 1.09-1.23) significantly influenced AKI development.

The magnitude of electrolyte-related risks differed substantially between sexes: hyperphosphatemia showed 2.7-fold stronger association in males. Conversely, lipid abnormalities demonstrated protective effects exclusively in females. Hypertension history uniformly increased risk in both sexes, while ST-elevation myocardial infarction (STEMI) reduced risk only in males (OR = 0.58). These differential associations highlight sex-specific pathophysiological mechanisms in AKI development following diabetic AMI.

### 3.4 Multivariable analysis of AKI risk factors

**Figure 3:**
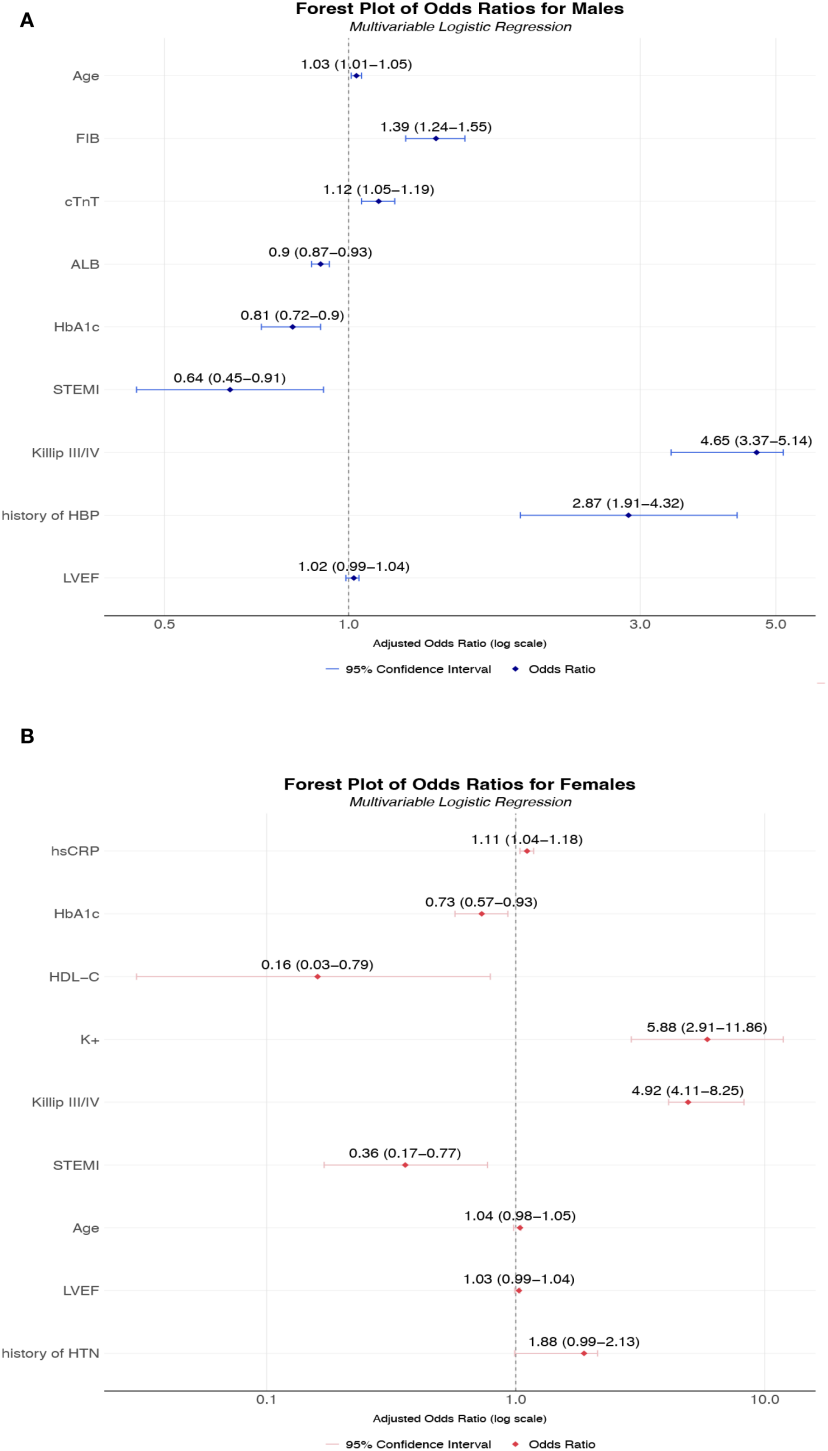
**(A)** Multivariable-adjusted odds ratios for AKI risk factors in male T2DM-AMI patients; **(B)** Multivariable-adjusted odds ratios for AKI risk factors in female T2DM-AMI patients. **(A)** A multivariable logistic regression model was used to analyze male patients with AMI and comorbid T2DM (n = 2002). Point estimates (squares) represent odds ratios (ORs), with horizontal lines indicating 95% confidence intervals. The vertical dashed line denotes the null effect (OR = 1). Key findings: Fibrinogen (FIB) significantly increased AKI risk (OR = 1.39, 95% CI 1.24-1.55). History of hypertension (HBP) showed the highest risk elevation (OR = 2.87, 95% CI 1.91-4.32). Albumin (ALB) demonstrated a protective effect (OR = 0.90, 95% CI 0.87-0.93). HbA1c and STEMI exhibited inverse associations (OR = 0.81 and 0.64, respectively). The model adjusted for confounders including age and cardiac injury biomarkers (cTnT). This multivariable model was adjusted for the following potential confounding factors: age, history of hypertension, and LVEF. **(B)** Multivariable logistic regression analysis of female patients with AMI and comorbid T2DM (n = 629). Squares represent point estimates (ORs), horizontal lines indicate 95% confidence intervals, and the vertical dashed line denotes the null effect (OR = 1). Key findings: Hyperkalemia (K^+^) markedly increased AKI risk (OR = 5.88, 95% CI 2.91-11.86). Killip class IV showed the highest risk (OR = 7.49, 95% CI 1.59-35.32). High-density lipoprotein (HDL-C) demonstrated strong protection (OR = 0.16, 95% CI 0.03-0.79). STEMI and HbA1c exhibited protective associations (OR = 0.36 and 0.73, respectively). Model adjusted for inflammatory markers including high-sensitivity C-reactive protein (hsCRP). This multivariable model was adjusted for the following potential confounding factors: age, history of hypertension, and LVEF. *All continuous variables were standardized; ORs correspond to per-standard-deviation changes. FIB, Fibrinogen; Hb1Ac, Hemoglobin A1c; HDL-C, High-Density Lipoprotein Cholesterol; hsCRP, High-Sensitivity C-Reactive Protein; K^+^, Potassium ion; ALB, Albumin; HTN, Hypertension; LVEF, left ventricular ejection fraction.

Multivariable logistic regression analysis identified markedly divergent AKI risk profiles between sexes (**Figure 3**). Among males, after adjusting for factors including age, history of hypertension, heart failure, and LVEF, significant independent predictors included advancing age (OR 1.03, 95% CI 1.01-1.05 per year), elevated fibrinogen (OR = 1.39, 95% CI 1.24-1.55), and hypertension history (OR = 2.87, 95% CI 1.91-4.32), while hypoalbuminemia (OR = 0.90, 95% CI 0.87-0.93) and STEMI presentation (OR = 0.64, 95% CI 0.87-0.93) conferred protection. Paradoxically, higher HbA1c reduced AKI risk (OR = 0.81, 95% CI 0.72-0.90). In females, risk associations demonstrated greater magnitude, with hyperkalemia emerging as the strongest predictor (OR = 5.88, 95% CI 2.91-11.86), followed by advanced Killip classification (Class III: OR = 5.99, 95% CI 1.63-21.93; Class IV: OR = 7.49, 95% CI 1.59-35.32). Notably, lipid metabolism exerted unique protection in females, with elevated HDL-C substantially lowering AKI risk (OR = 0.16, 95% CI 0.03-0.79), while STEMI (OR = 0.36, 95% CI 0.17-0.77) and higher HbA1c (OR = 0.73, 95% CI 0.57-0.93) exhibited unexpectedly protective effects. These results collectively demonstrate fundamentally distinct pathophysiological pathways for AKI development in T2DM- AMI patients according to sex.

## 4 Discussion

The robust sex disparities observed in acute kidney injury (AKI) risk among diabetic acute myocardial infarction (AMI) patients necessitate a fundamental reevaluation of pathophysiological paradigms. In this study, the incidence of AKI in patients with AMI Ccomplicated by T2DM was 13.3%, which is consistent with previous studies (17, 18). Our findings reveal three fundamental paradigms: (1) Female patients experience disproportionately higher AKI incidence (17.2% vs. 12.1%, *P* = 0.001) despite exhibiting protective metabolic features; (2) AKI risk factors demonstrate striking sexual dimorphism, with electrolyte imbalances dominating male susceptibility while lipid metabolism paradoxically protects females; and (3) Multivariable models confirm sex-stratified pathophysiological pathways, necessitating precision management approaches. These observations challenge the current “one-size-fits-all” AKI prevention strategies in diabetic AMI.

The elevated AKI risk in females—despite younger biological age typically conferring renal resilience—constitutes a critical finding. This phenomenon may stem from the “diabetic erosion” of estrogenic protection: while estrogen enhances endothelial NO synthase activity and promotes vasodilation under physiological conditions, chronic hyperglycemia in T2DM induces advanced glycation end-product accumulation that blunts these benefits (19, 20). Our data support this mechanism, with females exhibiting significantly higher HbA1c (8.31% vs. 8.13%, *P* = 0.012) and more severe insulin resistance. Concurrently, menopause-related hormonal shifts (mean female age = 72.4 years) exacerbate renal vulnerability through reduced renal plasma flow and upregulated RAAS activity (21), aligning with their higher hypertension prevalence (71.7% vs. 60.4%, *P* < 0.001). The 42% increased AKI risk in females underscores the inadequacy of current gender-neutral risk stratification tools, which overlook these pathophysiological nuances.

The extreme male susceptibility to hyperphosphatemia (OR = 14.32 vs. female OR = 5.24; *P* < 0.001) reveals a previously underappreciated electrolyte-axis dichotomy. Phosphorus depletion in diabetic males may trigger mitochondrial dysfunction in proximal tubules—already compromised by ischemic injury—through impaired ATP regeneration. This is compounded by androgen-driven upregulation of sodium-phosphate cotransporters (NaPi-IIa), increasing renal phosphate wasting during stress states (22, 23). Conversely, hyperkalemia emerged as the predominant female risk (OR = 5.88), likely reflecting their higher rates of RAAS inhibitor use (unadjusted in our model) and diminished renal functional reserve (lower baseline eGFR: 79.62 vs. 89.22 mL/min/1.73m²). These findings necessitate sex-tailored electrolyte monitoring: aggressive phosphate control in males versus stringent potassium control in females during AMI management.

The most unexpected finding was the robust AKI protection conferred by low HDL-C (OR = 0.16) and LDL-C (OR = 0.56) exclusively in females. It reveals critical divergence that HDL-C exerts exceptionally potent protection in females, nearly threefold stronger than LDL-associated risks. This might stems from estrogen-HDL synergy—17β-estradiol enhances capacity of HDL to shuttle cholesterol to adrenal glands via SR-B1 receptors, while upregulating endothelial lipase production that generates sphingosine-1-phosphate (S1P) (24, 25). Estrogen-HDL synergy enhances S1P-mediated glycocalyx integrity (via SR-B1/Rac1), reducing podocyte injury – a pathway blunted by testosterone in males. The resulting S1P gradient preserves glycocalyx integrity through Rac1-mediated cytoskeletal stabilization, reducing albuminuria and podocyte injury. In males, however, testosterone suppresses hepatic ApoA-I synthesis, diminishing antioxidative capacity of HDL against phospholipid peroxidation in renal microvasculature. Furthermore, this contradicts conventional cardiorenal doctrine but aligns with emerging evidence of HDL-mediated anti-inflammatory signaling in tubular cells (26). We posit that in the diabetic milieu, glycated HDL particles may paradoxically enhance cytoprotective effects via SR-B1 receptor activation, suppressing TNF-α–induced apoptosis. Supporting this, females demonstrated lower hsCRP (3.39 vs. 3.72 mg/L, *P* = 0.001)—a marker attenuated in our multivariate model. Mechanistically, estrogen modulates hepatic APOA1 synthesis, potentially generating renoprotective HDL subspecies. This paradigm-shifting observation warrants investigation into HDL composition rather than concentration as a novel therapeutic target.

The inverse association between elevated HbA1c levels and reduced risk of acute kidney injury (AKI) observed in this study appears to contradict the established pathophysiology linking hyperglycemia to microvascular injury. Rather than suggesting a protective effect of higher glycemia, this counterintuitive finding may largely arise from methodological biases, particularly reverse causation. Artificially lowered HbA1c values—resulting from anemia, erythrocyte turnover alterations, or malnutrition in patients with undetected advanced kidney dysfunction—could misclassify high-risk individuals into the low HbA1c group, thereby amplifying AKI risk in that subgroup. Survivor bias may also contribute, as patients with long-standing severe hyperglycemia and significant end-organ damage may have been excluded from the cohort, leaving a more resilient high-HbA1c group with intrinsically lower susceptibility to AKI. Furthermore, residual confounding related to differential clinical management or unmeasured factors such as body composition and nutritional status cannot be ruled out. Therefore, these results should not be interpreted as evidence of a beneficial effect of hyperglycemia, but rather underscore the complexity of interpreting observational data in the context of metabolic and renal disease. Future studies incorporating longitudinal HbA1c measurements, nutritional biomarkers, and detailed medication histories are warranted to clarify the true relationship between glycemic control and AKI risk.

Our regression models provide sex-specific AKI prediction frameworks: Male-predominant model: Driven by fibrinogen (OR = 1.39) and hyperalbuminemia (OR = 0.90), reflecting a prothrombotic-inflammatory axis. Fibrinogen directly promotes glomerular microthrombosis, while hypoalbuminemia exacerbates oxidant stress via diminished fatty acid binding capacity. Female-predominant model: Centered on Killip class (OR = 5.99-7.49) and hyperkalemia, indicating hemodynamic vulnerability. This correlates with their higher cardiogenic shock rates (Killip IV: 8.6% both sexes, but females had 47% higher stage 2–3 AKI). The paradoxical HbA1c protective effect (male OR = 0.81; female OR = 0.73), which may reflect unmeasured factors (e.g., antidiabetic regimens) requiring future pharmacovigilance studies.—acute hyperglycemia during AMI could mask chronic control benefits. Alternatively, higher HbA1c might select for residual beta-cell function, preserving renal autoregulation.

T2DM occurring in over 30% of AMI patients (27), significantly amplifies AKI risk through interconnected pathological mechanisms distinct from non-diabetic cohorts. Hyperglycemia induces mitochondrial dysfunction in proximal tubules via PARP-1 overactivation, depleting cellular ATP reserves and impairing tubular repair while advanced glycation end products disrupt podocyte integrity through RAGE receptor binding, collectively compromising renal function. These processes are exacerbated by diabetic dysautonomia, which blunts hemodynamic adaptation during cardiogenic shock through impaired renal sympathetic regulation. Crucially, T2DM modulates AKI risk through fundamentally sex-dimorphic pathways.

Therefore, these findings suggest the existence of a “cardio-renal sex paradox,” wherein female sex is associated with lower troponin release yet higher NT-proBNP and greater AKI risk following STEMI. Mechanistically, this may reflect sex-divergent pathophysiological pathways: in females, microvascular dysfunction may predominantly contribute to renal injury, possibly exacerbated by renal medullary hypoxia. Estrogen-mediated enhancement of endothelial progenitor cell recruitment might attenuate overt cardiomyocyte necrosis, yet remain insufficient to protect the renal microvasculature. These observations highlight the need for further investigation into the underlying mechanisms of sex-specific cardio-renal interactions in acute ischemia.

In summary, the convergence of female sex, advanced age and poor glycemic control defines a high-risk AKI phenotype in T2DM-AMI patients, driven by cardio-renal crosstalk and estrogen-deficient tubular vulnerability. To advance precision nephroprotection, we advocate for sex-specific AKI risk scores, pre-emptive renal protocols for elderly diabetic women, and mechanistic studies on estrogen’s role in renal glucose toxicity. Recognizing sex as a biological variable is not merely equitable—it is essential for optimizing outcomes in diabetic cardiovascular disease.

Several limitations should be acknowledged in interpreting our findings. Its observational design renders it susceptible to residual confounding and selection bias, particularly due to the absence of systematically collected data on urinary biomarkers and detailed medication adherence—factors that may affect AKI risk stratification. Although the substantial sample size enhances internal validity, we were unable to account for certain potential unmeasured confounders, such as medication adherence and detailed clinical criteria for dialysis initiation, because of the retrospective nature of our single-center study. The absence of these data introduces the possibility of residual confounding, which may have influenced the observed associations. The non-randomized treatment allocation further precludes definitive causal inferences regarding the observed sex-specific mechanisms. Furthermore, although our current study findings reveal important sex-specific clinical associations, these results are still limited by the inherent constraints of an observational study design. These limitations include the potential for unmeasured confounding factors, the inability to fully establish causal relationships, and the fact that the underlying biological pathways remain to be fully elucidated. To strengthen causal inference in future research, we plan to employ the target trial emulation (TTE) framework (28), which explicitly defines treatment strategies, eligibility criteria, time-zero, and outcomes—augmented with methods such as cloning and weighting—to mitigate biases and produce more reliable estimates. To conduct causal mediation analysis within the framework of structural equation modeling (SEM), a prospective design incorporating serial biomarker measurement data is required. This ensures temporally consistent modeling and analysis of exposure factors, mediator variables, and outcome indicators. Additionally, we will pursue prospective multicenter validation of risk models using machine learning; investigate hormonal dynamics (e.g., estradiol-to-testosterone ratios) in AK pathogenesis; and explore lipidomic signatures underlying HDL’s renoprotective effects via targeted mass spectrometry. While this study identifies significant associations at the population level, the averaging of effects across a diverse cohort may mask important variation among subgroups defined by clinical, biochemical, or pathophysiological characteristics. This is particularly relevant in the context of sex-specific risk patterns, where hormonal, genetic, or comorbidity-related factors may modify both risk and treatment response. Future studies should employ unsupervised learning methods—such as latent class analysis, Gaussian mixture models, or deep clustering—to identify subpopulations with shared biomarker patterns or risk trajectories (29). Our study exclusively included hospitalized inpatients, which introduces a potential for selection bias. This design necessarily captures a population with likely more severe disease and complications, potentially under-representing individuals with milder AMI managed in outpatient settings.

## Data Availability

The raw data supporting the conclusions of this article will be made available by the authors, without undue reservation.
